# Compositions and Functions of Mitochondria-Associated Endoplasmic Reticulum Membranes and Their Contribution to Cardioprotection by Exercise Preconditioning

**DOI:** 10.3389/fphys.2022.910452

**Published:** 2022-06-06

**Authors:** Yuhu Lv, Lin Cheng, Fenglin Peng

**Affiliations:** College of Physical Education and Health, Guangxi Normal University, Guilin, China

**Keywords:** mitochondria-associated endoplasmic reticulum membranes, exercise preconditioning, cardioprotection, exercise, preconditioning, heart

## Abstract

Mitochondria-associated endoplasmic reticulum membranes (MAMs) are important components of intracellular signaling and contribute to the regulation of intracellular Ca^2+^/lipid homeostasis, mitochondrial dynamics, autophagy/mitophagy, apoptosis, and inflammation. Multiple studies have shown that proteins located on MAMs mediate cardioprotection. Exercise preconditioning (EP) has been shown to protect the myocardium from adverse stimuli, but these mechanisms are still being explored. Recently, a growing body of evidence points to MAMs, suggesting that exercise or EP may be involved in cardioprotection by modulating proteins on MAMs and subsequently affecting MAMs. In this review, we summarize the latest findings on MAMs, analyzing the structure and function of MAMs and the role of MAM-related proteins in cardioprotection. We focused on the possible mechanisms by which exercise or EP can modulate the involvement of MAMs in cardioprotection. We found that EP may affect MAMs by regulating changes in MFN2, MFN1, AMPK, FUNDC1, BECN1, VDAC1, GRP75, IP3R, CYPD, GSK3β, AKT, NLRP3, GRP78, and LC3, thus playing a cardioprotective role. We also provided direction for future studies that may be of interest so that more in-depth studies can be conducted to elucidate the relationship between EP and cardioprotection.

## Introduction

Myocardial infarction (MI) is an acute coronary syndrome of cardiovascular diseases (CVDs) with high lethality. It is a progressive factor in heart failure (HF) and myocardial cell death, leading to myocardial damage and cardiac insufficiency (Yellon & Hausenloy, 2007). Myocardial ischemia/reperfusion (I/R) injury (IRI) is frequently considered to be one of the most serious risk factors for coronary artery disease, which has been attributed primarily to oxidative stress and endoplasmic reticulum (ER) stress (ERS), as well as to mitochondrial dysfunction (Bi et al., 2018). Mitochondrial oxidative phosphorylation impairment, reactive oxygen species (ROS) production, permeability transformation, and Ca^2+^ overload-induced swelling are all associated with mitochondrial dysfunction (Bernardi, 2013). Ca^2+^ overload leads to dissipation of the mitochondrial membrane potential and subsequent opening of the mitochondrial permeability transition pore (mPTP) (Ong et al., 2015), which leads to IRI. During I/R, Ca^2+^ enters the mitochondria through an associated influx mechanism leading to ROS accumulation, which initiates the opening of mPTP and induces cell death (Bernardi, 2013). Thus, Ca^2+^ transfer between the ER/sarcoplasmic reticulum (SR) and mitochondria is an essential part of the occurrence of CVDs.

ER and mitochondria are two critical organelles that play an essential role in cellular protein production and energy metabolism. In addition, they are vital organelles associated with Ca^2+^ homeostasis, working together and simultaneously establishing a balance between Ca^2+^ release and cytoplasmic buffering (Erustes et al., 2021). One of the chief roles of ER is ensuring the folding of the polypeptide chains transferred from the cytoplasm and transporting the resulting proteins to the Golgi apparatus (Pinton, 2018). The ER is responsible for the folding of one-third of human proteins and is coordinated by the protein disulfide (PDI) family of ER oxidoreductases, some of which are also known to participate in Ca^2+^ transport (Carreras-Sureda et al., 2018). In addition, the ER is the primary site of lipid synthesis, membrane biogenesis, and exogenous detoxification. As a result, the normal functioning of mitochondria and ER is essential for maintaining the homeostasis of the intracellular environment (Sun and Ding, 2020). Unlike the single membrane of the ER, mitochondria consist of two layers—the outer mitochondrial membrane (OMM) and the inner mitochondrial membrane (IMM)—that fold to form ridges for the attachment of aerobic oxidases. The IMM is beneficial for maintaining the morphology and function of mitochondria within normal ranges for many physiological processes in the cell.

Recent studies have shown that mitochondria also can interact with the ER, lipid droplets, Golgi apparatus, lysosomes, and peroxisomes to form temporal and spatial interorganelle connections that play an instrumental role in normal cellular function (Keenan et al., 2020). Csordás et al. (2018) proposed the concept of intercellular contacts to explain these phenomena, arguing that intercellular contacts are central to the control of cellular behavior. The ER and mitochondria are not isolated, but rather are connected by tethered complexes called mitochondria-ER connections/interactions/contacts or mitochondria-associated (ER) membranes (MAMs). MAMs are the areas in the ER where lipid rafts are closely associated with mitochondrial and multiple enzyme activities converge to coordinate cellular functions (Merle et al., 2019). MAMs are “liquid-ordered” lipid phases in the ER characterized by high molecular packing, low diffusivity, and saturated lipid acetyl chains (Montesinos and Area-Gomez, 2020). As the name suggests, MAMs include OMM and ER, and contain lipids, tethered proteins, and calcium transport proteins that play important roles in cellular energy metabolism, Ca^2+^/lipid homeostasis, mitochondrial dynamics, ER redox control, autophagy, apoptosis, and inflammatory responses (Yan et al., 2017; Xiao et al., 2020).

The concept of ischemic preconditioning (IP) was first introduced in 1986 by Murry et al. (1986), who found that multiple anginal episodes before MI may delay cell death after coronary occlusion, resulting in greater myocardial salvage during reperfusion therapy. The concept of exercise preconditioning (EP) was introduced by Yamashita et al. (1999), has effects similar to those of IP, and acts in cardioprotection (Quindry & Franklin, 2021). EP can be divided into early EP (EEP) and late EP (LEP), both of which have a protective effect on myocardial injury, and it has been found that EEP and LEP also can have a synergistic effect, thus enhancing the protection of the heart by EP (Liu and Pan, 2019). It also has been shown that EP can affect MAMs by regulating MAM-resident proteins and thus exert a protective effect on the myocardium. Through simplified preclinical studies, cardioprotective mechanisms of EP have been demonstrated, including upregulation of endogenous antioxidant enzyme activity, improved calcium handling, and enhanced regulation of bioenergetics during supply-demand mismatch (Quindry & Franklin, 2021). In this review, we explored which MAM-resident proteins can be altered by exercise or EP. We comprehensively combined the structure and function of MAMs and identified possible therapeutic targets for related CVDs by analyzing the potential mechanisms of EP cardioprotection. This work supports the development of new strategies for the treatment of CVDs.

## Composition and Structure of MAMs

### Isolation of MAMs

The composition of MAMs is highly conserved across species and tissues (Luan et al., 2021). Proteins residing in MAMs are part of the physical interactions of MAMs or are involved in the regulation of complexes in MAMs (Lan et al., 2019). Because the density of the MAMs region is lower than that of all the ER, many protocols take advantage of its low density to isolate it (Sala-Vila et al., 2016; Schreiner & Ankarcrona, 2017; Montesinos & Area-Gomez, 2020). Therefore, MAMs are susceptible to contamination by low-density membranes, such as lysosomes, endosomes, and peroxisomes. Montesinos and Area-Gomez’s scheme can be used to obtain MAMs, they used continuous density sucrose gradients combined with detergent treatment to separate the MAM fraction from the other small membrane compartments (Montesinos & Area-Gomez, 2020). Analysis of organelle interactions by multispectral image acquisition can overcome the challenge of spectral overlap in fluorescent protein palettes (Valm et al., 2017).

### Structure and Compositions of MAMs

The membrane connection between ER and mitochondria is referred to as the ER-mitochondrial encounter structure (ERMES) or the ER membrane protein complex (EMC). The MAM interface consists of phospholipid bilayers composed of ER and mitochondria, with smooth and rough ER-mitochondrial contacts at distances of 10–50 and 50–80 nm, respectively (Sukhorukov et al., 2022). Researchers initially believed that MAMs were less than 100 nm wide (Zhang et al., 2021). Kornmann et al. (2009) screened mutants that artificially designed synthetic proteins could supplement and found that the Mmm1/Mdm10/Mdm12/Mdm34 complex was a molecular chain between the two organelles ER-mitochondria, which promoted calcium and phospholipid exchange between them.

The MAMs of human and mouse testis have more than 1,000 highly conserved proteins (Wang et al., 2018). Using electron microscopy and fluorescence-based organelle proximity probes, Naon et al. (2016) demonstrated that ER-mitochondrial juxtaposition was reduced by the absence of mitofusin 2 (MFN2), an ER-mitochondrial-tethering protein whose ablation reduces intercellular juxtaposition and communication. They also found that ablation of MFN2 reduced mitochondrial calcium uptake induced by inositol-1,4, 5-triphosphate (IP3), which led to significant morphological changes in the ER (Naon et al., 2016). MFN2 in ER is attached to MFN1 or MFN2 on mitochondria, forming homotypic (MFN2-MFN2) or heterotypic (MFN2-MFN1) complexes that link two adjacent organelles, and ablation of MFN2 may loosen ER-mitochondrial interactions (Yu et al., 2018). Another study showed that acute downregulation of MFN2 increased ER and mitochondrial contacts (Cieri et al., 2018). It is unclear whether the specific actions of MFN2 in SR/SR-mitochondria were positive or negative because the changes in mitochondrial morphology caused by MFN2 ablation complicated the ER/SR-mitochondrial interface (Yu et al., 2018). Presenilin 2 (PS2) also regulates ER-mitochondrial coupling, but it can exert its antagonistic effect through regulation only in the presence of MFN2. Furthermore, homologs of MFN2 and PS2 (MFN1 and PS1) are indispensable in this physical interaction (Filadi et al., 2016). These results suggest that the presence of MFN2 in MAMs may provide conditions for other molecules to play regulatory roles.

**FIGURE 1:**
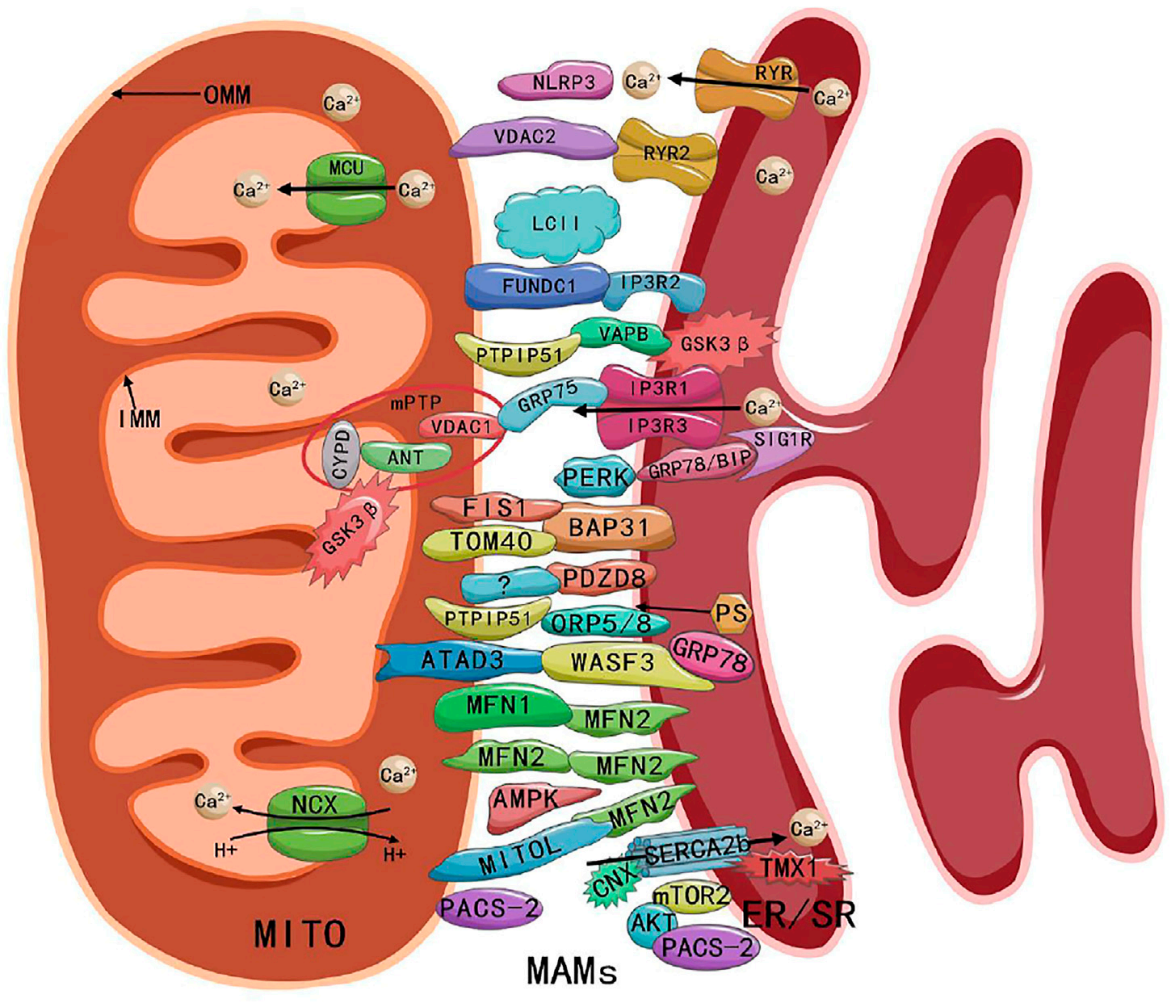
Composition and structure of MAMs. Abbreviations: MITO, mitochondria; MAMs, mitochondria-associated endoplasmic reticulum membranes; ER/SR, endoplasmic/sarcoplasmic reticulum; OMM, outer mitochondrial membrane; IMM, inner mitochondrial membrane; MCU, mitochondrial Ca^2+^ uniporter; NCX, Na^+^ Ca^2+^ exchanger; RYR, ryanodine receptor; NLRP3, NOD-like receptor protein 3; VDAC, voltage-dependent anion channel; FUNDC1, FUN14 domain containing 1; IP3R, inositol-1,4, 5-triphosphate receptor; PTPIP51, protein tyrosine phosphatase-interacting protein 51; VAPB, vesicle-associated membrane-protein-associated protein B; GSK3β, glycogen synthase kinase-3 beta; CYPD, cyclophilin D; ANT, adenine nucleotide transporter; mPTP, mitochondrial permeability transition pore; GRP75/78, glucose-regulated protein 75/78; SIG1R, sigma-1 receptor; PERK, PRK-like ER kinase; FIS1, fission 1; BAP31, B cell receptor-associated protein 31; TOM40, outer mitochondrial membrane 40; PDZD8, PDZ domain-containing protein 8; ORP5/8, oxysterol-binding protein-related protein 5/8; PS, phosphatidylserine; ATAD3, ATPase family AAA domain-containing protein 3; WASF3, Wiskoff-Aldrich syndrome protein family member 3; MFN1/2, mitofusin 1/2; AMPK, AMP-activated protein kinase; CNX, Calnexin; SERCA, Sarco/ER Ca^2+^ ATPase; TMX1, thioredoxin-related transmembrane protein 1; PACS-2, phosphofurin acidic cluster sorting protein 2; AKT, protein kinase B; mTORC2, mammalian TOR complex 2.

Sun and Ding (2020) suggested that MAMs consist of a number of essential proteins, such as MFN1, MFN2, IP3 receptor (IP3R), glucose-regulated protein 75 (GRP75), voltage-dependent anion channel (VDAC), protein tyrosine phosphatase-interacting protein 51 (PTPIP51), B-cell receptor-associated protein 31 (BAP31), mitochondrial fission 1 (FIS1), vesicle-associated membrane-protein-associated protein B (VAPB), and phosphofurin acidic cluster sorting protein 2 (PACS-2). MAMs also include regulatory proteins, such as Sarco/ER Ca^2+^ ATPase (SERCA), Calnexin (CNX), phosphatidylserine synthase (PSS), long-chain fatty acid coenzyme A (CoA) ligase 4 (FACL4), sigma-1 receptor (SIG1R), ER oxidoreductin-1α (ERO1α), autophagy-related gene (ATG14), cyclophilin D (CYPD), protein kinase B (AKT), and mammalian TOR complex 2 (mTORC2). These above proteins work individually or by linking to form complexes (e.g., MFN2-MFN1/2 complex, IP3R-GRP75-VDAC1 complex, PTPIP51-VAPB complex, and BAP31-FIS1 complex) (Sun & Ding, 2020). The ATPase family AAA domain-containing protein 3 (ATAD3) proteins (ATAD3A and ATAD3B) regulate MAMs by interacting with GRP78/BiP-Wiskoff-Aldrich syndrome protein family member 3 (WASF3) complex, in which WASF3 acts as a bridge and has been identified in breast cancer cells (Baudier, 2018). Endogenous PINK1 and Beclin1 (BECN1) also have been found to be relocalized in MAMs during mitosis or autophagy (Gelmetti et al., 2017). A recent study showed that PDZ domain-containing protein 8 (PDZD8) was an important ER-mitochondrial-tethering protein in metazoans and that PDZD8 is required for ER-mitochondrial contact formation in mammalian cells (Hirabayahi et al., 2017). The presence of the NOD-like receptor protein 3 (NLRP3) inflammasome in MAMs also has been detected (Missiroli et al., 2018). Ryanodine receptor 2 (RYR2) -VDAC2, SERCA-thioredoxin-related transmembrane protein 1 (TMX1), glycogen synthase kinase-3 beta (GSK3β), mitochondrial Ca^2+^ uniporter (MCU), microtubule-associated protein 1 light chain 3 (LC3) (Yuan et al., 2018), and FUN14 domain containing 1 (FUNDC1) also have been detected in cardiomyocyte MAMs (Nikolaou et al., 2019; Sun et al., 2019; Wu & Zou, 2019; Xiao et al., 2020; Gong et al., 2021; Jiang et al., 2021; Luan et al., 2021). The compositions of MAMs as shown in Figure 1.

## Function of MAMs

The first report on the function of MAMs was in 1967 (Fleischer et al., 1967). The biological function of MAMs is influenced significantly by the number, length, and distance of connections between the ER and mitochondria (Yu et al., 2021).

### Modulation of Ca^2+^ Homeostasis

Because Ca^2+^ is involved in a number of enzymes that regulate ATP synthesis and is involved in the production of ATP by mitochondria through the tricarboxylic acid cycle (Wu & Zou, 2019), maintaining relatively constant levels of Ca^2+^ is essential for proper cellular functioning. Three main cardiac Ca^2+^ regulatory proteins *in vivo* (i.e., Na^+^-Ca^2+^ (NCX) exchanger proteins, SERCA, and RYR Ca^2+^ release channel proteins) are involved in regulating [Ca^2+^]i (Bupha-Intr & Wattanapermpool, 2006). IP3R and SERCA are two crucial ER-related Ca^2+^ handling proteins in MAMs. IP3Rs can interact with VDAC1 and act as a bridge between ER with OMM with the molecular chaperone GRP75, forming the IP3Rs-GRP75-VDAC1 complex responsible for Ca^2+^ transfer from ER to OMM (Yu et al., 2021). OMM transports Ca^2+^ into the membrane gap through VDAC1. Unlike OMM, IMM is not permeable to Ca^2+^, and Ca^2+^ within the membrane gap enters the mitochondrial matrix via MCU (Wu and Zou, 2019). The IP3Rs-GRP75-VDAC1 complex is regulated by IRE1α, DJ-1, FUNDC1, and other substances that affect the formation of MAMs, which in turn regulate Ca^2+^ release from the ER to the mitochondria and cytoplasm in mouse cardiomyocytes (Wu et al., 2017; Carreras-Sureda et al., 2019; Liu and Pan, 2019). Another study showed that MFN2 is thought to act as an antagonist of ER and mitochondrial association, preventing excessive and potentially toxic proximity of both organelles and that ablation of MFN2 increases ER-mitochondrial coupling, thereby enhancing IP3-induced Ca^2+^ transfer from ER to mitochondria and making cells more sensitive to mitochondrial Ca^2+^ overload-dependent death (Filadi et al., 2015). In addition, VAPB located on the ER and PTPIP51 located on the OMM can form a VAPB-PTPIP51 complex, which also is involved in Ca^2+^ regulation between the ER and mitochondria (Gómez-Suaga et al., 2019). Alpha-synuclein (α-syn) is also a cell membrane protein found to be located at MAMs enrichment sites. Although it is not a mitochondrial targeting protein, it does interact with VAPB, thereby inhibiting the joint action of VAPB and PTPIP51 (Guardia-Laguartaet al., 2014). SERCA2b is enriched in MAMs and is a subtype of SERCA, showing the highest affinity for Ca^2+^. CNX and TMX1 interact directly with SERCA2b to modulate SERCA2b activity in a palmitoylation-dependent manner. Palmitoylated CNX can increase SERCA2b activity, thereby reducing Ca^2+^ flux between ER-mitochondria, whereas TMX1 counteracts CNX-SERCA2b coaction, inhibits SERCA2b activity, and promotes Ca^2+^ influx into mitochondria (Gutiérrez and Simmen, 2018). In neurons, PDZD8 has been shown to be involved in the regulation of Ca^2+^ homeostasis, and PDZD8 also induces the release of Ca^2+^ from the synaptic endoplasmic reticulum, which is later taken up by mitochondria, thereby regulating cytoplasmic Ca^2+^ dynamics (Hirabayahi et al., 2017).

### Modulation of Lipid Homeostasis

Lipids are involved in many cellular physiological processes, including participation in building cell membranes and in energy metabolism. The tether proteins of MAMs, including cholesterol acyltransferase/sterol O-acyltransferase 1 (ACAT1/SOAT1), FACL4, diacylglycerol O-acyltransferase 2 (DGAT2), PSS, and phosphatidylethanolamine N-methyltransferase 2 (PEMT2), may be involved in the synthesis and transport of phospholipids and function as a platform for lipid biosynthesis and exchange (Anastasia et al., 2021; Luan et al., 2021). FACL4 regulates the binding of fatty acids to CoA and other cholesterol metabolites and often is considered to be a standard marker for the identification of MAMs (Yu et al., 2021). In addition to being involved in calcium homeostasis, PTPIP51 can be tethered to oxysterol-binding protein-associated protein 5/8 (ORP5/8) on the ER membrane to facilitate phosphatidylserine (PS) transfer to mitochondria (Luan et al., 2021).

A liver study showed that hormone-induced and ATAD3-mediated formation of MAMs is involved in the optimal transfer of cholesterol from the ER to the mitochondria, leading to steroidogenesis (Issop et al., 2015). Caveolin-1 (CAV-1) is a pivotal cholesterol efflux regulator enriched in the domain of MAMs, which plays a central role in the recruitment and regulation of cholesterol and steroids. Its genetic defect can lead to reduced physical extension and integrity of MAMs as well as to the irregular accumulation of free cholesterol (Sala-Vila et al., 2016). Another study showed that decreased skeletal muscle CAV-1 and elevated cholesterol led to oxidative stress and mitochondrial dysfunction (Flis et al., 2018). MFN2 has been shown to mediate the interaction between mitochondria and lipid droplets, affecting lipolysis and systemic energy homeostasis (Boutant et al., 2017). Loss or gain of GRP75 and MFN2 function can significantly alter cholesterol metabolism (Bassot et al., 2021). Knapp et al. (2019) demonstrated that adhesion-G-protein-coupled receptors (ADGRs) play a role not only in synapses, but also in the ER, Golgi apparatus, mitochondria, and MAMs. Knapp et al. (2019) used proteomic data obtained by tandem affinity purifications combined with mass spectrometry and demonstrated that SIG1R is a core protein of MAMs. SIG1R also may be involved in the regulation of lipid rafts (Zhang et al., 2020). It was shown that in the ER, PDZD8 binds to Protrudin as well as Rab7 endosomes and recruits mitochondria to form three-way contacts and that PDZD8 mediates the lipid transfer process (Guillen-Samander et al., 2019; Elbaz-Alon et al., 2020; Shirane et al., 2020; Gao et al., 2022). These studies have demonstrated that MAMs constitute an important platform for lipid homeostasis and are involved in the regulation of lipid homeostasis.

### Modulation of Mitochondrial Dynamics

Mitochondrial dynamics include mitochondrial fusion, fission, and motility. Mitochondrial fusion promotes the exchange of internal material between mitochondria, thereby allowing damaged mitochondria to recover. MFN in OMM and optic atrophy 1 (OPA1) in IMM are the two proteins that are primarily responsible for regulating mitochondrial fusion. The ubiquitination of MFN2 by mitochondrial ubiquitin ligase (MITOL) or the phosphoric ubiquitination of MFN2 by Parkinson protein 2 E3 ubiquitin-protein ligase (Parkin) and PTEN-induced kinase 1 (PINK1) reduces the stringing of ER-mitochondria. In addition, MFN2 tethering with MITOL also regulates mitochondrial dynamics (Sugiura et al., 2013; McLelland et al., 2018). mTORC2-AKT-mediated phosphorylation of PACS-2 has been shown to affect the integrity of MAMs (Betz et al., 2013). Ablation of MFN2 or depletion of PACS-2 may lead to ER fragmentation or mitochondrial fragmentation (Sun & Ding, 2020).

Several proteins in OMM, including mitochondrial fission factor (Mff), FIS1, and mitochondrial dynamics 51/49 kDa protein (MiD51/MiD49, also known as MIEF1/MIEF2) could recruit GTPase dynamicity-associated protein 1 (DRP1) to promote mitochondrial fission (Zhang et al., 2016; Yu et al., 2017). Syntaxin 17 (STX17), inverted formin 2 (INF2), and RAB32, which are involved in the regulation of mitochondrial fission, also have been detected in MAMs (Luan et al., 2021). Mitochondrial fission is induced in MAMs, during which DRP1 is recruited to the OMM and forms a helix around the OMM, contracting and splitting the mitochondria into two parts. In addition to recruiting DRP1 to the mitochondrial fission sites, FIS1 also interacts with BAP31 to induce apoptosis (Iwasawa et al., 2011). BAP31 regulates mitochondrial function by interacting with the OMM 40 (TOM40) translocase at the ER-mitochondrial contact site (Namba, 2019).

ER-mitochondrial contact is not a seamless connection between ER and mitochondria. The spacing between the two organelles allows for mitochondrial motility, and excessive ER-mitochondrial contact and Ca^2+^ transfer might give rise to defects in the mitochondrial transport axis (Modi et al., 2019). MAMs may provide a better platform for mitochondrial Rho GTPase 1 (MIRO1) and MIRO2 to fine-tune actin-dependent and tubulin-dependent mitochondrial motility and localization to regulate critical cellular functions (such as cell proliferation) (Castro et al., 2018).

### Modulation of Autophagy, Mitophagy, Apoptosis, and Inflammation

Autophagy, first discovered and named in 1967, is a process of homeostasis and self-renewal by lysosomal degradation of damaged organelles and macromolecules (Dete and De Duve, 1967). Appropriate levels of autophagy are protective against noxious stimuli, whereas excessive levels can have detrimental effects (Liu et al., 2022). A previous study demonstrated that autophagosomal membranes may be derived from MAMs (Hamasaki et al., 2013). This research has shown that MAMs can provide membranes for autophagosome formation, that disruption of MAMs with depletion of MFN2 significantly attenuates autophagy, and that mitochondria also play a vital role in autophagy (Gomez-Suaga et al., 2017; Manganelli et al., 2021). A study found that the AMP-activated protein kinase (AMPK)-MFN2 axis regulates energy stress-induced dynamics of MAMs and autophagy by establishing a molecular link between AMPK and MFN2 (Hu et al., 2021). A recent study showed that reduced expression of MFN2-MFN1, VAPB-PTPP51, and BAP31-FIS1 in MAMs could lead to abnormal hippocampal autophagy in rats (Liu et al., 2022). The VAPB-PTPIP51 tether regulates autophagy by mediating the transfer of Ca^2+^ from ER storage to mitochondria. Overexpression of VAPB or PTPIP51 increases damage to MAMs, whereas deletion of VAPB or PTPIP51 mediated by siRNA attenuates MAMs and promotes autophagosome formation (Gomez-Suaga et al., 2017). PACS-2 also has been shown to regulate autophagosome formation in MAMs, and inhibition of PACS-2 expression reduces the starvation-induced autophagy marker LC3II in cells (Hamasaki et al., 2013; Zhang et al., 2021b). Another study showed that both endogenous PINK1 and BECN1 were found to relocalize in MAMs during mitosis or autophagy, promoting tethering of ER to mitochondria and autophagosome formation (Gelmetti et al., 2017).

An *in vitro* experiment showed that HK-2 cells overexpressing PACS-2 alleviated hyperglycemia-induced mitochondrial fission by blocking DRP1 recruitment in mitochondria, thereby restoring the integrity of MAMs and enhancing mitochondrial phagocytosis. Mechanistically, PACS-2 binds to BECN1 and mediates the relocalization of BECN1 to MAMs, promoting the formation of mitophagosome (Li et al., 2022). MAMs are destroyed during mitophagy and reducing MAMs increases the rate of mitochondrial degradation because MFN2 promotes mitophagy by activating p97-dependent ER release in mitochondria through PINK1/Parkin ubiquitination (McLelland et al., 2018). FUNDC1 is involved in mitophagy, and FUNDC1-mediated mitophagy plays an essential role in the remodeling of the mitochondrial network in the differentiation of adult cardiac-specific progenitor cells (Lampert et al., 2019; Liu et al., 2021).

MAMs are physiological regulators of apoptosis, modulating the process of apoptosis (Coku et al., 2022). MAMs that modulate the crosstalk between mitochondria and ER can regulate ERS and mitochondria-mediated apoptosis (Wang et al., 2022). A previous study has shown that GRP75 plays a vital role in mediating palmitate-induced ER-mitochondrial interactions leading to islet cell apoptosis (Tiwary et al., 2021). In addition, a study in diabetic mice found that PACS-2 knockout (KO) resulted in disruption of MAMs, mitochondrial dysfunction, increased kidney cell apoptosis, and fibrosis (Xue et al., 2021). The FIS1-BAP31 complex across the interface of MAMs provides a platform for activating procaspase-8, thereby linking the apoptotic signaling of two essential organelles (Iwasawa et al., 2011). PRK-like ER kinase (PERK) is an abundant component of MAMs that interacts with multifunctional MAM-tethering proteins to integrate and regulate the exchange of contact point substances (e.g., lipids, Ca^2+^, and ROS), PERK also transmits signals from the nucleus to these organelles with membrane structures, forming an interconnected network that regulates 17β-estradiol-induced apoptosis (Fan and Jordan, 2022). MAMs are essential molecular platforms for the formation of the NLRP3 inflammasome, a site of MAMs that rapidly senses the extent of the injury and coordinates an appropriate response. NLRP3 deficiency can attenuate cardiomyopathy by inhibiting mitochondrial dysfunction (Missiroli et al., 2018).

## Roles of MAM-Related Proteins in Cardioprotection

### CYPD

Traditionally, mPTP consists of an adenine nucleotide transporter (ANT) located in the IMM, a VDAC located in the OMM, and CYPD within the matrix, of which CYPD is the principal regulator of mPTP (Elrod and Molkentin, 2013). During I/R, the interaction between CYPD and the VDAC1-GRP75-IP3R1 complex is strengthened, delivering mitochondrial Ca^2+^ overload and cardiomyocyte death. Furthermore, deletion of the CYPD-encoding gene PPIF in cardiomyocytes disrupts the synergistic interaction between CYPD and IP3R1 and reduces hypoxia and reoxygenation (HR)-induced mitochondrial Ca^2+^ overload (Paillard et al., 2013). Similarly, inhibition of CYPD interactions with N-methyl-4-isoleucine cyclosporine or the IP3R1 inhibitor 2-aminoethoxydiphenylboronate protects against myocardial calcium overload or death, whereas downregulation of CYPD or IP3R1 can achieve similar effects (Paillard et al., 2013). It has been suggested that reducing the contact between SR and ER may protect the myocardium from IRI caused by calcium overload. Results showed that hypoxic preconditioning (HP) or IP protected the myocardium from I/R-induced cell death in the presence of CYPD, whereas it did not in the case of CYPD deficiency, and the same result occurred when CYPD was inhibited by cyclosporine A (Hausenloy et al., 2010). Nevertheless, another study showed that cardiac-specific ablation of IP3Rs in mice did not show a significant reduction in I/R injury compared with wild type (Garcia and Boehning, 2017). Parkin inhibits the opening of mPTP by catalyzing the ubiquitination of CYPD in the necrotic cascade, inhibiting necrosis, reducing myocardial IRI, and improving cardiac function. These cascade responses, however, are not involved in Parkin-regulated mitophagy (Sun et al., 2019). Thus, inactivation or ablation of CYPD, the IRI-implementing molecule, ameliorates myocardial injury by inhibiting mitochondrial Ca^2+^ overload. At the same time, the protective effect of preconditioning was lost in *Ppif* ^*−/−*^ mice, which were more susceptible to HF in the presence of stress overload (Lim et al., 2007). Thus, CYPD may play a double-edged role in I/R (Gong et al., 2021).

### GSK3β

GSK3β, an essential regulator of Ca^2+^ transfer in cardiomyocytes, interacts with IP3R1-GRP75-VDAC1 in a constitutively active form at the site of MAMs. The inhibitor SB216763 inhibits GSK3β activity, not only impairing the interaction of IP3R1-GRP75-VDAC1, but also reducing Ca^2+^ transfer from SR to mitochondria (Gomez et al., 2016). During hypoxia and reoxygenation, SB216763 prevents the rise in IP3R1 activity and prevents mitochondrial Ca^2+^ overload by reducing GSK3β-mediated phosphorylation of IP3R1 (Gomez et al., 2016). Inhibition of GSK3β activity with MLS2776 and MLS2778 resulted in smaller infarct areas in rabbits and mice than in controls, and pharmacological inhibition of GSK3β was a viable strategy for limiting infarct area independent of cyclosporine A-induced mPTP inhibition (Nikolaou et al., 2019). Meanwhile, targeting VDAC1 with small interfering RNA (siRNA) reduces GSK3β translocation to mitochondria and prevents the turn-on of mPTP in the cellular stress response (Tanno et al., 2014). GSK-3 deletion, however, induces mitotic mutations in adult cardiomyocytes, accompanied by an increase in DNA levels and nuclei, and leading to apoptosis and fatal diabetic cardiomyopathy (DCM) (Zhou et al., 2016). Phosphorylation of GSK3β at Tyr216 increased its activity, whereas phosphorylation of GSK3β at Ser9 inhibited its activity and protected the heart during IP (Tong et al., 2002). Phosphorylation of GSK3β at Ser9 also can bind to ANT and competitively inhibit CYPD binding, inhibiting the opening of mPTP (Gomez et al., 2008).

### VAPB and PTPIP51

A study on neuronal cells showed that increased activation of GSK3β in malignant fusions led to the segregation of the MAMs scaffold complex VAPB-PTPIP51 and a reduction in ER-mitochondrial contacts, resulting in impaired mitochondrial function (Stoica et al., 2016). Disruption of VAPB and PTPIP51 coupling is associated not only with ER-mitochondrial segregation but also with disruption of Ca^2+^ exchange and energy production (Paillussonet al., 2017). PTPIP51 is significantly upregulated in I/R hearts, and adenovirus-mediated overexpression of PTPIP51 significantly increases SR-mitochondrial contacts. In addition, unidirectional MCU increases Ca^2+^ uptake, and inhibition or knockdown of MCU reverses the PTPIP51-mediated increase in mitochondrial Ca^2+^ and protects cardiomyocytes from PTPIP51-mediated apoptosis. Similarly, cardiac PTPIP51-specific knockdown significantly reduces nodule size and cardiac injury after myocardial infiltration of I/R, suggesting that PTPIP51 could be a therapeutic target for ischemic heart disease (Qiao et al., 2017). Downregulation of VAPB or PTPIP51 can activate autophagy through the downregulation of mitochondrial Ca^2+^ levels (Gomez-Suaga et al., 2017), but the mechanism of PTPIP51 in the regulation of reperfusion injury remains unclear.

### MFN1 and MFN2

A related study showed that mitochondria take up 1–15% of cytoplasmic Ca^2+^ during the heartbeat, and the rest is taken up by SERCA and NCLX. The lack of NCLX in the adult mouse heart led to sudden death, and mitochondrial Ca^2+^ overload led to severe myocardial dysfunction and fulminant HF (Drago et al., 2012). In contrast, overexpression of NCLX in mouse hearts enhanced mitochondrial Ca^2+^ clearance and protected the myocardium from ischemia-induced cellular necrosis and HF (Luongo et al., 2017). The conditional KO technique Mer-Cre-Mer induced an increased resistance to myocardial IRI in MFN2 or MFN1 KO mice (Papanicolaou et al., 2011; Hall et al., 2016). Cardiac MFN2-specific KO mice, however, exhibited mild hypertrophy and impaired cardiac function with less severe symptoms than MFN1 and MFN2 double-KO mice (Hall et al., 2016), suggesting that the tasks of MFN1 and MFN2 could be analogous. The deletion of MFN1 and MFN2 led to ER-mitochondrial uncoupling, thereby reducing the sensitivity to mimic I/R-induced mitochondrial Ca^2+^ overload, ROS production, and mPTP opening (Papanicolaou et al., 2011). In addition, deletion of MFN2 led to both increased distance between mitochondria and ER and separation of CYPD and IP3R1-GRP75-VDAC complex, further reducing reperfusion injury (Paillard et al., 2013). Another study, however, showed that ablation of MFN2 increased ER-mitochondrial coupling and enhanced IP3-induced Ca^2+^ transfer from ER to mitochondria, making cells sensitive to mitochondrial Ca^2+^ overload-dependent death, and MFN2 was suggested to be an antagonist of ER-mitochondrial junctions, which prevented excessive and potentially toxic proximity between the two organelles (Filadi et al., 2015). In addition, another study showed that four-month-old mice did not show any improvement in cardiac function over controls after myocardial-specific ablation of MFN2, whereas six-month-old mice showed an increase in I/R damage (Zhao et al., 2012). Overall, short-term deficiency of MFN1/2 may alleviate cardiac insufficiency, as inhibition of mitochondrial Ca^2+^ overload and oxidative respiratory chain activity during reperfusion may attenuate oxidative stress (Ong et al., 2014). Long-term MFN2 deficiency, however, will induce cardiac dysfunction because of impaired autophagy and reduced mitochondrial fusion (Zhao et al., 2012). In addition, MFN2-deficient neonatal rat cardiomyocytes are more prone to H_2_O_2_ damage and mitochondrial depolarization (Papanicolaou et al., 2011), which may be related to the different dependence of cardiomyocytes on MFN2 at different developmental stages and the functional diversity of MFN1/2 (Gong et al., 2021).

### FUNDC1

FUNDC1, a protein located on the OMM, is involved in the formation of MAMs by binding to IP3R2, maintaining the dynamics and function of cardiac mitochondria *in vivo*. Transmission electron microscopy showed that deletion of FUNDC1 resulted in approximately 80% reduction in ER and mitochondrial contacts, whereas deletion of MFN2 caused only approximately 30% reduction (Chen et al., 2012; Wu et al., 2017). FUNDC1 improved IP3R2 stability by directly interacting with IP3R2 and inhibiting its ubiquitination and degradation. Mutant FUNDC1 was not able to bind to IP3R2, and significantly reduced contact between the ER and mitochondria, whereas a similar situation was seen after FUNDC1 ablation. Cardiac mitochondria in FUNDC1-specific KO mice were larger and longer. A significant decrease in ventricular filling rate, ejection fraction, and cardiac output in early and late stages led to myocardial systolic and diastolic dysfunction (Wu et al., 2017). Results also showed that in septic mice, upregulation of FUNDC1-dependent formation of MAMs could lead to increased occurrence of cardiac dysfunction (Jiang et al., 2021). Meanwhile, inhibition of mTORC1-ULK1-FUNDC1 pathway-mediated mitophagy protects the myocardium from IRI (Xiao et al., 2020). Another study came to the opposite conclusion, however, indicating that inhibition of FUNDC1-induced mitophagy promoted IRI (Yu et al., 2019). FUNDC1-mediated mitophagy may play a pro-survival role when damaged mitochondria are removed and newly generated mitochondria can be replenished. It also may promote cell death when mitophagy exceeds the cellular tolerance and newly generated mitochondria cannot be replenished to meet cellular needs. The exact mechanism needs to be further investigated.

### SIG1R and BECN1

Sigma receptors are nonopioid receptors located at the ER, including at least two subtypes (SIG1R and SIG2R). SIG1R ensures stable Ca^2+^ signal transduction between the ER and mitochondria by maintaining IP3R stability (Hayashi & Su, 2007). Upregulation of SIG1R in rats plays a role in maintaining cardiac function (Lou et al., 2021). SIG1R inhibition accelerates autophagy in cardiomyocytes in oxidative stress conditions (Ortíz-Rentería et al., 2018). Particular SIG1R agonists modulate the contractility of cardiomyocytes. In addition, SIG1R activation inhibits angiotensin II-induced myocardial hypertrophy and myocardial cell damage. SIG1R KO mouse showed mitochondrial dysfunction, morphological abnormalities, and cardiac remodeling, leading to systolic dysfunction (Luan et al., 2021). Upregulated SIG1R can preserve heart function in rats with MI (Lou et al., 2021). Another study showed that genetically or pharmacologically targeted activation of BECN1 improved MAMs in the heart during endotoxemia, suggesting that BECN1 may also be involved in cardiac protective processes. (Sun et al., 2021).

## EP-Mediated Cardioprotection and its Possible Mechanisms With MAM-Related Proteins

### EP-Mediated Cardioprotection May be Achieved by Modulating MFN2 and MFN1

Both MFN1 and MFN2 are involved in mediating mitochondrial fusion and mitophagy and have been found to accumulate significantly in the failing heart. Eight weeks of exercise reduced the accumulation of MFN1 and MFN2 in the failing heart, reestablished mitochondrial fission–fusion homeostasis, and reduced the accumulation of mitochondrial debris, thus providing cardioprotection (Campos et al., 2017). The PINK1/Parkin pathway of mitophagy can be activated by ubiquitination or phosphorylation of MFN2 (Masaldan et al., 2022). In 2018, Pan’s lab demonstrated that PINK1-mediated mitophagy played an essential role in LEP-induced cardioprotection (Yuan et al., 2018). Another study showed that, although exercise did not significantly alter the change of MFN2, it could attenuate negative mitochondrial biogenesis regulation by enhancing the MFN2/Drp-1 ratio in the db/db mice hearts (Veeranki et al., 2016). A six-week exercise intervention significantly reduced p-Drp1^Ser616^/Drp1 levels and increased MFN2/VDAC levels in the heart induced by a high-fat diet (Palee et al., 2019). From these studies, it is clear that MFN2 or MFN1 may be involved in EP-mediated cardioprotection, but more direct pieces of evidence are needed to demonstrate how exercise modulates the involvement of MFN2 or MFN1 in EP-mediated cardioprotection, and the mechanisms behind this need to be urgently addressed.

### EP-Mediated Cardioprotection May be Achieved by Modulating AMPK and FUNDC1

A recent study showed that under energy stress, as mitochondrial fission occurs, a large amount of AMPK is transferred from the cytoplasm to the MAMs and mitochondria and interact directly with MFN2 (Hu et al., 2021). Swimming training attenuates isoproterenol-induced myocardial fibrosis by inhibiting AMPK activation-mediated NADPH oxidase-ROS pathway, suggesting that cardioprotection developed through exercise training is AMPK-dependent (Ma et al., 2015b). Liu et al. demonstrated that LEP-mediated myocardial protection is achieved by activating the AMPK-mTOR-ULK1 pathway to promote autophagy (Liu and Pan, 2019). The study showed that at different times of MI activation of autophagy and apoptosis through activation of the AMPK-mTOR signaling pathway within 6 h of ischemia could synergistically exert myocardial protective effects. Results also showed that the expression of p-AMPK showed a time-dependent trend, showing that p-AMPK levels increased at 0 h after MI, decreased at 1 h, gradually peaked at 6 h, and decreased again at 12 h, whereas p-mTOR levels showed a continuous decrease over time and autophagy-related protein LC3 was continuously upregulated (Ma et al., 2015). By performing hypoxia/reoxygenation (H/R) simulated I/R using H9c2 cells placed *in vitro*, it was found that myocardial ischemic post-processing promoted autophagy in response to IRI by activating the nNOS/AMPK/mTOR pathway (Hao et al., 2017). An intensity effect provided by exercise also may promote myocardial protection. Prolonged high-intensity exercise training causes myocardial hypertrophy with cardiac injury and risks pathological changes. The mTOR signaling pathway may play a critical regulatory role in myocardial hypertrophy after prolonged moderate exercise, but not after high-intensity exercise (Liao et al., 2015). Studies have shown that cardioprotective effects can be achieved by modulating FUNDC1 (Yu et al., 2019; Memme et al., 2021). Empagliflozin protects mitochondrial function and attenuates cardiac microvascular I/R injury by activating the AMPKα1/ULK1/FUNDC1/mitophagy pathway (Cai et al., 2022). A study of electroacupuncture preconditioning showed that electroacupuncture preconditioning attenuated myocardial IRI by inhibiting mTORC1-ULK1-FUNDC1 pathway-mediated mitophagy (Xiao et al., 2020). HP induces FUNDC1-dependent mitochondrial phagocytosis in platelets and reduces I/R-induced cardiac injury (Zhang et al., 2017). Direct evidence demonstrating that exercise mediates cardioprotection through modulation of FUNDC1 is still lacking, and future studies in this area will be interesting.

### EP-Mediated Cardioprotection May be Achieved by Modulating BECN1 and VDAC1

As previously described, PINK1 and BECN1 relocalize on MAMs during mitochondrial autophagy, facilitating the association between ER and mitochondria and the formation of autophagosomes (Gelmetti et al., 2017). IPC inhibits BECN1-dependent excessive autophagy during the reperfusion phase and cooperates with the antiapoptotic pathway to reduce myocardial IRI-induced cell death (Peng et al., 2013). Results showed that EP-induced ischemic hypoxia is a crucial factor in BECN1-dependent autophagy-mediated cardioprotection (Yuan et al., 2020). Another study showed that EP stimulated an increase in BECN1 through intermittent myocardial ischemia and hypoxia, induced a protective effect of autophagy in the myocardium, and attenuated EE-induced myocardial ischemic-hypoxic injury (Huang et al., 2021). EEP significantly enhanced oxidative protection and inhibited EE damage by enhancing BECN1-dependent autophagy and BNIP3-dependent mitophagy. EE also can provide limited mitochondrial protection by inhibiting VDAC1 but not mitochondrial autophagy (Yuan et al., 2018). VDAC1 and IP3R1 form a VDAC1-GRP75-IP3R1 complex through the GRP75 bridge and are regulated by CYPD. Few studies, however, have examined the effect of exercise on this complex in the heart, and additional studies are necessary to elucidate the mechanism of EP cardioprotection.

### EP-Mediated Cardioprotection May Be Achieved by Modulating GSK3β and AKT

EP promoted the activation of the phosphorylated GSK3β kinase pathway, which reduced oxidative stress, inflammatory cytokines, and apoptosis and also increased the bioavailability of serum NO in I/R-injured rats, thus exhibiting cardioprotective effects (Sharma et al., 2018). Increased GSK3β activity and IP3R phosphorylation during hypoxic reperfusion were associated with cell death and increased the transfer of calcium ions from the SR/ER to mitochondria. Inhibition of GSK3β during reperfusion reduced phosphorylation of IP3R and Ca^2+^ release from SR/ER, which in turn reduced cytoplasmic and mitochondrial Ca^2+^ concentrations and susceptibility to apoptosis, suggesting that GSK3β inhibition during reperfusion reduced Ca^2+^ leakage from IP3R in cardiac MAMs, thereby limiting cellular and mitochondrial Ca^2+^ overload and subsequent cell death and acting as a cardioprotective agent (Gomez et al., 2016). Results showed that pGSK3β signaling during exercise training was protective against myocardial injury in isproterenol-treated rats, whereas quantitative estimation of total phosphorylated AKT^Ser473^/GSK3β^Ser9^ in isproterenol-treated animals showed a significant decrease in pAKTand pGSK3β (Sharma et al., 2018b). An 8-week aerobic exercise study showed protection against myocardial IRI, including antiapoptosis, at least in part through PI3K-dependent and AKT-mediated mechanisms (Zhang et al., 2007). AKT signaling, which can be inhibited by DCM, was found to be significantly activated by 15 weeks of exercise and mediated the cardioprotection against DCM. In the late stages of DCM, exercise maintained cardiac function, prevented cardiomyocyte apoptosis and fibrosis, and improved mitochondrial biogenesis (Wang et al., 2015). HP has the same protective effect on cardiac progenitor cells through the PI3K/AKT pathway (Xu et al., 2016).

Exercise plays a crucial role in cardioprotection via the IGF1-PI3K-AKT signaling pathway in transgenic mouse models of acquired and lost stimulation (Weeks et al., 2017). EP regulates the Bcl-2 family, reduces mPTP opening levels, protects the heart from EE-induced damage, reduces cardiomyocyte apoptosis, and improves cardiac function, which also has been associated with upregulation of the PI3K-AKTsignaling pathway (Li et al., 2020). Even after MI, 4 weeks of exercise training significantly increased the levels of NAD-dependent deacetylase sirtuin1 (SIRT1) and peroxisome proliferator-activated receptor γ coactivator 1α (PGC-1α). Training also increased p-PI3K and p-AKT levels by enhancing SIRT1/PGC-1α/PI3K/AKT signaling adaptive activation and acting as a protective agent for myocarocytes (Jia et al., 2019). Remote ischemic preconditioning-mediated cardioprotection also is associated with PI3K/AKT-dependent signaling pathways (Yu et al., 2014). Although this evidence has suggested that AKT is involved in EP-mediated cardioprotection, direct evidence showing that exercise affects MAMs by modulating AKT (and thus MAMs) is still lacking. Future studies are necessary to elucidate the effects of AKT on MAMs and its role in myocardial protection.

### EP-Mediated Cardioprotection May be Achieved by Modulating NLRP3, GRP78, and LC3

Currently, the NLRP3 complex remains the only inflammasome complex found to be associated with MAMs (Missiroli et al., 2018). Results have shown that acute exercise-induced mitochondrial stress enhanced ROS production and triggered the rat myocardial inflammatory response through NLRP3 inflammasome activation, but also increased activation of mitophagy to reduce myocardial injury (Li et al., 2016). Although acute exercise increased the expression of thioredoxin-interacting protein (TXNIP), nuclear transcription factor kappa B_p65_ (NF-κB_P65_), NLRP3, and Caspase-1, EP at different intensities protected the heart from EE-induced injury by downregulating the TXNIP/thioredoxin protein (TRX)/NF-κB_P65_/NLRP3 inflammatory signaling. EP at moderate intensities had the best protective effect (Li et al., 2020b). Exercise also may exert cardioprotective effects by modulating GRP78. Research has shown that cardiac dysfunction in MI rats is consistent with increased protein levels of GRP78, which is one of the unfolded protein response markers; accumulation of misfolded and polyubiquitinated proteins; and reduced chymotrypsin-like proteasome activity. Aerobic exercise training attenuates MI-induced ERS by reducing protein levels of unfolded protein response markers, including GRP78, and accumulation of misfolded and polyubiquinated proteins, which in turn has a cardioprotective effect (Bozi et al., 2016). From this analysis, it is clear that EP exerts cardioprotective effects through the regulation of NLRP3 and GRP78, seemingly through their modulation of other pathways. It would be interesting to investigate their direct role in exercise in the future.

Autophagosomal membranes may be derived from MAMs (Yang et al., 2020). LC3 is recruited to OMM during autophagy, and EEP exerts cardioprotective effects by recruiting LC3 to induce mitophagy (Yuan et al., 2018). The LC3II/LC3I ratio increased significantly at 2 h during the cardioprotective phase after EP (Li et al., 2019). The mechanism may be that EP induces myocardial autophagy through intermittent ischemia-hypoxia, increases LC3 lipidation-related proteins, and promotes the formation of autophagosome, thereby exerting cardioprotective effects (Li et al., 2019; Wan et al., 2021). In contrast, another study showed that EP reverses EE-induced autophagy by reducing autophagosome and LC3II turnover in cardiomyocytes and attenuating myocardial injury (Lu et al., 2018). These conflicting results suggested that EP-induced autophagy may play a dual role, in which increasing autophagy within the tolerable range of the heart or cardiomyocytes may be beneficial for cardioprotection and beyond that may have the opposite effect. More evidence is needed to support this idea.

## Conclusion

**FIGURE 2:**
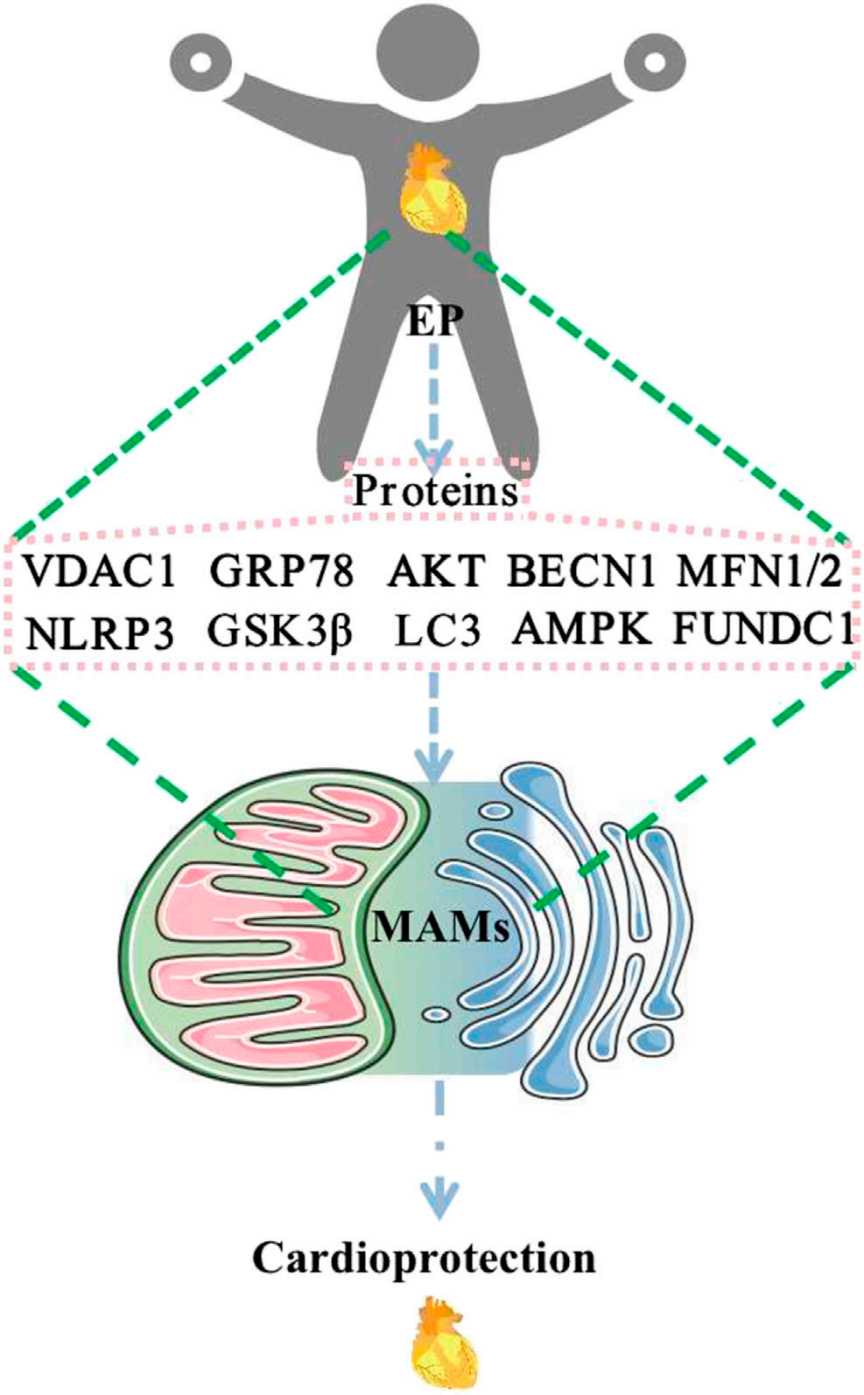
EP regulates MAM-related proteins to promote cardioprotection. Abbreviations: EP, exercise preconditioning; MAMs, mitochondria-associated endoplasmic reticulum membranes; VDAC1, voltage-dependent anion channel 1; GRP78, glucose-regulated protein 78; AKT, protein kinase B; BECN1, beclin1; MFN1/2, mitofusin 1/2; NLRP3, NOD-like receptor protein 3; GSK3β, glycogen synthase kinase-3 beta; LC3, microtubule-associated protein 1 light chain 3; AMPK, AMP-activated protein kinase; FUNDC1, FUN14 domain containing 1.

Available evidence has suggested that numerous proteins are involved in the composition of MAMs and may be differentially expressed in different tissues and organs. MAMs have functions in regulating Ca^2+^ homeostasis, mitochondrial dynamics, lipid homeostasis, ERS, autophagy, mitophagy, apoptosis, and inflammation. EP may play a role in cardioprotection through the regulation of MFN2, MFN1, AMPK, FUNDC1, BECN1, VDAC1, GRP75, IP3R, CYPD, GSK3β, AKT, NLRP3, GRP78, and LC3 (Figure 2). These changes affect MAMs, which mediate cardioprotection. Regulatory approaches that target these proteins may help develop new strategies for therapeutic intervention in various human diseases, including cardiovascular disease. At this point, EP or exercise remains the most effective behavioral therapy for improving heart health. Furthermore, studying exercise patterns that are both simple and modulate these target proteins would help address the issue of exercise adherence. Direct evidence of EP modulation of MAMs is scarce, and relevant investigators can increase studies in this direction to provide more reliable evidence to elucidate the mechanism of EP-mediated cardioprotection. In addition, MAMs are based on lipid membranes, and it is also necessary for researchers to increase studies on the relevant lipid components in MAMs, which seems to be a research gap. Increasing studies in this area will provide a brighter prospect for elucidating the structure and function of MAMs.
